# Evidence of HIV-1 Genital Shedding after One Year of Antiretroviral Therapy in Females Recently Diagnosed in Bamako, Mali

**DOI:** 10.3390/microorganisms9102164

**Published:** 2021-10-17

**Authors:** Abdelaye Keita, Josselin Rigaill, Sylvie Pillet, Youssouf Sereme, Souleymane Coulibaly, Fodé Diallo, Paul Verhoeven, Bruno Pozzetto, Tenin Aoua Thiero, Thomas Bourlet

**Affiliations:** 1 Département Qualité Sécurité et Sûreté Biologique, Institut National de Recherche en Santé Publique (INRSP), Bamako BP 1771, Mali; abdelaye@gmail.com (A.K.); sbcoulibaly1.sc@gmail.com (S.C.); teninaoua@yahoo.fr (T.A.T.); 2 GIMAP Team 15, Inserm, U1111, CNRS, UMR5308, Centre International de Recherche en Infectiologie, Faculty of Medicine, University of Lyon 1, 42000 Saint-Etienne, France; josselin.rigaill@chu-st-etienne.fr (J.R.); sylvie.pillet@chu-st-etienne.fr (S.P.); seremeyoussouf@yahoo.fr (Y.S.); paul.verhoeven@chu-st-etienne.fr (P.V.); bruno.pozzetto@chu-st-etienne.fr (B.P.); 3 Laboratoire des Agents Infectieux et d’Hygiène, Biology Pathology Department, University Hospital of Saint-Etienne, 42000 Saint-Etienne, France; 4 Centre d’Ecoute de Soins et d’Accompagnement (CESAC), ARCAD/SIDA Clinic, Bamako BPE 2561, Mali; fodie10@yahoo.fr

**Keywords:** HIV-1, antiretroviral therapy, genital reservoir, resistance, microbiota, Mali

## Abstract

Little is known about the dynamic of HIV-1 shedding and resistance profiles in the female genital reservoir after antiretroviral therapy (ART) initiation in resource-limited countries (RLCs), which is critical for evaluating the residual sexual HIV-1 transmission risk. The present study aimed to evaluate the efficacy of 1 year duration ART at blood and genital levels in females newly diagnosed for HIV-1 from three centers in Bamako, Mali. Seventy-eight consenting females were enrolled at the time of their HIV-1 infection diagnosis. HIV-1 RNA loads (Abbott Real-Time HIV-1 assay) were tested in blood and cervicovaginal fluids (CVF) before and 12 months after ART initiation. Primary and acquired resistances to ART were evaluated by Viroseq^TM^ HIV-1 genotyping assay. The vaginal microbiota was analyzed using IonTorrent^TM^ NGS technology (Thermo Fisher Scientific). Proportions of primary drug resistance mutations in blood and CVF were 13.4% and 25%, respectively. Discrepant profiles were observed in 25% of paired blood/CVF samples. The acquired resistance rate was 3.1% in blood. At month 12, undetectable HIV-1 RNA load was reached in 84.6% and 75% of blood and CVF samples, respectively. A vaginal dysbiosis was associated with HIV RNA shedding. Our findings emphasize the need of reinforcing education to improve retention in care system, as well as the necessity of regular virological monitoring before and during ART and of implementing vaginal dysbiosis diagnosis and treatment in RLCs.

## 1. Introduction

In 2016, the United Nations approved the 95/95/95 treatment targets with the aim of ending the HIV/AIDS epidemic by 2030 [1]. To this end, 95% of people living with HIV (PLWH), 95% of diagnosed people under combined antiretroviral treatment (cART), and 95% of people under treatment with fully suppressed viral load (VL) are the goals to reach. In this context, the initiation of cART in all PLWH at any CD4^+^ T-cell count has been recommended by the World Health Organization (WHO) since 2015 [2]. Thanks to this universal access to cART, AIDS-related mortality and new adult HIV-1 infections have declined dramatically during past years. Nevertheless, 1.7 million newly infected people were recorded in 2019. In sub-Saharan Africa, the epidemic is particularly ongoing in women who account for a disproportionate one in four sex ratio of new HIV infections among adults and adolescent girls. Women and girls accounted for 58% of the estimated 240,000 (150,000–390,000) new infections in 2019, reflecting the continuing role of gender inequalities in this part of the world. In addition, only 58% of PLWH were accessing antiretroviral therapy in 2019 [3]. Consequently, UNAIDS recently warned that progress in combating viral transmission is still not happening fast enough, with the risk that the 2020 targets of reducing AIDS-related deaths and new HIV infections to fewer than 500,000 will be missed.

Concomitantly to the ART scale-up in resource-limited countries (RLCs), the rates of viral failure (VF) and of HIV-1 pretreatment drug resistance mutations (PDRMs) tend to increase over time, especially toward first-generation non-nucleosidic reverse transcriptase inhibitors (NNRTIs) that are still widely used in first-line regimens [4,5,6].

It has been long recognized that cervicovaginal HIV shedding is associated with increased female-to-male and mother-to-child transmission [7]. As suggested by the results of the HPTN 052 study, ART can be expected to limit HIV transmission by reducing the concentration of virus in the blood and genital secretions of the infected person [7]. Several groups also reported a correlation between HIV-1 viral loads in blood and cervical secretions and a markedly reduced rate of transmission among persons with very low HIV viral loads in blood [8,9]. However, ongoing virus production in the genital tract of cART experienced females, who were virologically suppressed at the blood level, has been described, especially in the setting of local inflammation [10]. Numerous factors such as hormonal contraception, genital ulcerations or tenderness, high level of genital proinflammatory cytokines, vaginal dysbiosis, or sexually transmitted infections (STIs) could promote HIV-1 shedding at the genital level [11,12,13]. In particular, bacterial vaginosis due to common gastrointestinal tract commensals such as *Prevotella* spp. and *Gardnerella* spp. can induce genital inflammatory cytokine responses and alter the vaginal microbiome; consequently, antibiotics and/or probiotics are some of the new directions being pursued for HIV prevention [14,15].

In the era of the 95/95/95 plan, it is of high importance to monitor viral suppression and to check regularly the treatment cascade in PLWH. However, little is known about the dynamic of HIV-1 VL and resistance profiles in the genital reservoir after ART initiation in RLCs, which is critical for evaluating the residual risk of HIV-1 transmission via the sexual route in ART-experienced people [16].

In a previous study, we described a high prevalence of PDRMs in the blood of people newly diagnosed for HIV-1 in Bamako, Mali, associated with a reassuring virological success rate, as well as a low level of acquired mutations in cART-adherent people [17]. In the extension phase of this study, we aimed to evaluate the potential risk of HIV-1 transmission via genital secretions, especially in the context of an area with a high PDRM level, as well as to assess the efficacy of a 12 month cART regimen on HIV shedding in the genital tract of newly HIV-1-diagnosed females in Bamako.

## 2. Patients and Methods

### 2.1. Patients

A total of 78 newly diagnosed HIV-1 females were enrolled between January and June 2014 at 3 centers, namely, Centre d’Écoute, de Soins, et d’Accompagnement (CESAC), Commune 1, and ARCAD/SIDA clinic in Bamako. ART was initiated in all participants within 2 weeks of HIV diagnosis, combining either two nucleosidic reverse transcriptase inhibitors (NRTIs) and one NNRTI or two NRTIs and one protease inhibitor (PI) in 95.8% and 4.2% of patients, respectively. The most commonly used combination was zidovudine/3TC or FTC/efavirenz (85.6%). Whole blood and cervicovaginal fluid (CVF) were sampled at the time of HIV diagnosis in each female and at month 12 after ART initiation.

### 2.2. Samples

A fresh blood sample containing EDTA was used for CD4^+^ T-cell count. Blood plasma was obtained from a second whole-blood sample after centrifugation at 1500× *g* for 20 min. CVF was collected from the posterior fornix of the vagina by lavage with a volume of 10 mL of physiological saline solution. Blood plasma and CVF were stored at −80 °C until analysis. The presence of semen or blood in CVF was checked visually under microscopic examination (×40) by a laboratory technician at INRSP in Bamako.

### 2.3. CD4^+^ Cell Count

The CD4^+^ T cells were quantified by the BD FACSCount™ System (Becton–Dickinson, Franklin Lakes, NJ, USA) at INRSP in Bamako.

### 2.4. HIV RNA Viral Load

HIV-1 RNA was quantified in blood plasma and CVF by the Abbott Real-Time HIV-1 assay (Abbott Molecular, Rungis, France) at the Infectious Agents and Hygiene Laboratory of the University Hospital of Saint-Etienne. The cutoff value was 40 copies/mL.

### 2.5. Genotyping Resistance Assay

All genotyping resistance assays were done retrospectively at the end of the study on blood/CVF paired samples detected positive for HIV VL before ART start and 12 months after treatment initiation. Total RNA was extracted using the NUCLISENS easyMAG platform (bioMérieux, Marcy l’Etoile, France). The HIV-1 reverse transcriptase (RT) and protease genes were amplified and sequenced using the ViroSeq HIV-1 Genotyping System^®^ (Celera Diagnostics, City of Alameda, CA, USA). The resulting sequences were aligned using ViroSeq^®^ HIV-1 Genotyping System Software v2.6 (Celera Diagnostics). HIV-1 subtype was established from the RT/protease sequence analysis using the Stanford HIV Drug Resistance Database (https://hivdb.stanford.edu/hivdb/by-sequences/, accessed on 11 May 2021). Drug resistance mutations (DRMs) were defined as those appearing in the WHO SDRM list (available at https://hivdb.stanford.edu/page/who-sdrm-list/, accessed on 11 May 2021). The DRM interpretation was carried out according to the algorithm of the HIV db program, available at https://hivdb.stanford.edu/, accessed on 12 May 2021).

### 2.6. Phylogenetic Analysis

Protease and RT genes from patients and reference strains were aligned by using MUSCLE software (https://www.drive5.com/muscle/, accessed on 19 May 2021) with minor manual adjustments [18]. Reference strains were obtained from the Los Alamos HIV Sequence Databases (https://www.hiv.lanl.gov/components/sequence/HIV/search/search.html, accessed on 19 May 2021). Phylogenetic and molecular analyses were conducted using MEGA software (version 2.1, University Park, Philadelphia, PA, USA) by a neighbor-joining method with pairwise deletion [19]. The genetic distances were calculated using the maximum likelihood method [20]. The reliability of branching orders was assessed by a bootstrap method (1000 repetitions).

### 2.7. Nucleotide Sequence Accession Numbers

Nucleotide sequences were deposited in GenBank under the following accession numbers: MG838583 to MG838594, MG838596, MG838597, MG838600 to MG838615, MG838618 to MG838634, MG838637, MG838640 to MG838644, MG838648, MG838649, MG838651 to MG838655, MG838657 to MG838659, MG838661 to MG838664, MG838666 to MG838672, and MN273741 to MN273750.

### 2.8. Vaginal Microbiota Analysis 

After a proteinase K treatment (Eurobio Scientific, Les Ulis, France) for 1 h at 56 °C, total DNA was isolated using the QIAamp DSP DNA Mini Kit on a QIAsymphony platform (Qiagen, Courtaboeuf, France) according to the manufacturer’s instructions. After extraction, the 12 samples (from six patients, before and after HIV treatment) were amplified using the Platinum PCR SuperMix High Fidelity (Invitrogen, Villebon sur Yvette, France) and specific primers for the domain V3 of rDNA bacteria. The V3 region primers 338F (ACTCCTACGGGAGGCAGCAG) and 533R (TTACCGCGGCTGCTGGCAC) were modified by the addition of Personal Genome Machine (PGM) sequencing adaptors and barcodes [21,22]. PCR conditions consisted of an initial taq activation for 2 min at 94 °C followed by 50 steps of 20 s denaturation at 94 °C, 20 s annealing cycle at 64 °C, and 35 s extension cycle at 68 °C. Amplicons were purified using the GeneRead Size Selection Kit (Qiagen). The concentration of amplicons was then evaluated with the data High-Sensitivity DNA Kit (Agilent Technologies, Santa Clara, CA, USA). Ion 318 chip V2 was prepared using the Ion Chef System, and sequencing was performed on Ion PGM system using the Ion PGM Hi-Q View Chef 400 Kit (ion Torrent, ThermoFisher Scientific, Waltham, MA, USA). Sequences were analyzed using mothur v1.40.5 with operational taxonomic units (OTUs) defined at 95% sequence similarity using the 16 S Silva database v132. Sequences which did not respected the following criteria were discarded: length between 130 and 210 base pairs without primers, adaptors, and barcodes (195 base pairs expected on *E. coli* reference strain), <8 homopolymers, average quality score above 22, and sequences representing at least more than 0.5% of the number of trim sequences of a sample. Analyses were restricted to genus level due to the limitations of the resolution on taxonomical classification using 16 S gene sequencing. If needed, a representative sequence for an unidentified OTU was BLASTed against the NCBI 16 S ribosomal RNA sequences database for identification. Calculation of α-diversity was performed within the mothur pipeline.

### 2.9. Statistical Analysis

Continuous variables were compared using Student’s *t*-test, ANOVA analysis, and nonparametric Mann–Whitney test when required. Comparisons between categorical variables were done using the chi^2^ and Fisher’s exact tests when appropriate. The correlation between quantitative values was analyzed using the Spearman correlation test. A *p*-value < 0.05 was considered to be statistically significant.

## 3. Results

### 3.1. Characteristics of the Studied Population at the Time of Diagnosis

A total of 78 females were included in the study (mean age: 34 ± 9 years), 38.5% of them at an III/IV WHO clinical stage. This studied population was quite immunosuppressed, since the mean CD4^+^ T-cell count was 354 ± 319 cells/mm^3^ of blood, compared to the healthy Malian population (902 cells/mm^3^ (444–1669)), as reported by Kone et al. [23]. Samples could be collected prospectively over a period of 12 months in 52 out of 78 females (66.7%). Three women (3.8%) died during the study, and 23 (29.5%) were lost to follow-up (LTFU). A statistically significant association was observed between mortality and a low CD4^+^ T-cell count (200 cells/mm^3^), as well as a clinical stage of III/IV.

### 3.2. HIV-1 Viral Load in Blood and Cervicovaginal Fluids

The mean HIV-1 RNA load in blood was 173,000 ± 32,200 copies/mL. HIV RNA was detected positive in 84.6% of CVF samples (53,130 ± 13,800 copies/mL). The mean HIV RNA value was significantly higher in blood than in genital secretions (*p* < 10^−5^). HIV-1 RNA VL was not correlated between blood and CVF (*r* = 0.07; *p* = 0.57). CD4^+^ T-cell values were not negatively correlated with HIV-1 RNA VL in blood (*r* = −0.15; *p* = 0.20) or in genital secretions (*r* = −0.10; *p* = 0.10). 

### 3.3. HIV-1 Subtypes and Phylogenetic Analysis

**Figure 1:**
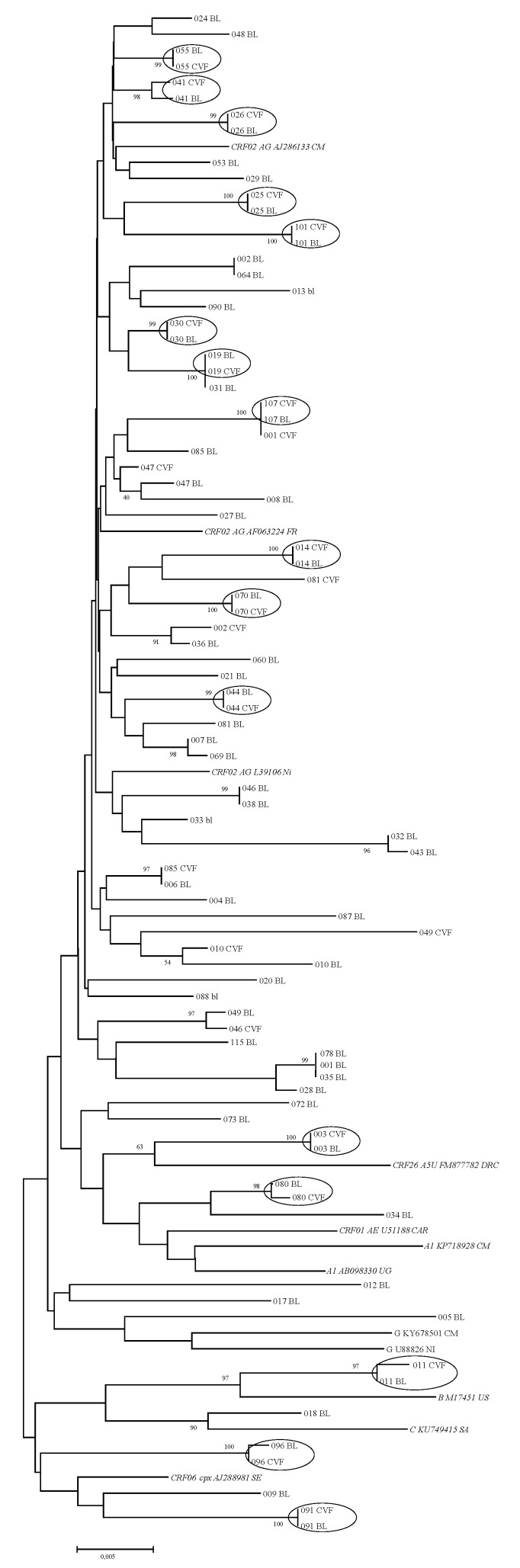
Phylogenetic tree based on reverse-transcriptase and protease genes of 84 samples obtained from blood (BL, *n* = 60) and cervicovaginal fluid (CVF, *n* = 24). Strains from the same patient exhibiting identical sequences are shown in a circle (bootstrap values above 96%).

Sixty complete RT/protease genes were successfully sequenced out of the 78 blood samples at the time of HIV-1 diagnosis. The most prevalent subtype was CRF02_AG (78.3%), followed by CRF06_cpx (6.7%), A1 (5%), CRF26_A5U (3.3%), C (3.3%), G (1.7%), and B (1.7%). Twenty-four sequences were also obtained from cervicovaginal secretions. A phylogenetic tree was constructed with these 84 RT/protease sequences (Figure 1). Sixteen paired sequences from samples of blood and genital secretions exhibited high rates of homology, ranging from 97% to 100%. Eight other paired sequences exhibited more discrepant homology identity percentages between blood and CVF, ranging from 90.9% to 95.2%.

### 3.4. Pretreatment Drug Resistance Mutations in Blood and CVF

Among the 60 RT/protease gene sequences obtained from blood, eight exhibited at least one mutation conferring resistance against NNRTI, NRTI, or PI in blood, which corresponds to a PDRM rate of 13.4%. The PDRM percentages for NRTIs, NNRTIs, and PIs were 5% (3/60), 16.6% (10/60), and 0%, respectively. The most prevalent NRTI resistance mutation was M184V (3.3%) followed by T215Y (1.7%). The following NNRTI resistance mutations were detected: V179I (8.3%), K103N (6.7%), V106I (5%), E138A (3.3%), Y181C (3.3%), and K103E (1.7%). In addition, the V179E nonpolymorphic mutation was found in four patients (6.3%). The following polymorphic mutations were also detected: L10I/V and K20I mutations on all tested protease sequences; V90I and V179T on three (5%) and three (4.8%) reverse-transcriptase genes. Two patients exhibited mutations conferring resistance to both NRTI and NNRTI drugs.

Reverse transcriptase and protease genes sequences were successfully obtained from 24 CVF (Table 1). Six of them (25%) exhibited PDRMs, namely, L74V (one), D67N (two), E138A (one), V106I (one), and V179E (one). The proportions of PDRMs in CVF samples for NRTIs, NNRTIs, and PIs were 12.5%, 12.5%, and 4.2%, respectively.

Considering paired sample results, discrepant patterns were observed in six cases (25%). In four of them, PDRMs were present in CVF but not in the corresponding blood, namely, L74V (one), D67N (two), V106I (one), and V179E (one). Conversely, one K103N and one V179E mutation were detected in blood but not in the paired CVF sample. No presence of sperm cells or blood was visualized in these samples. As a whole, in the 24 females sampled at both blood and genital levels, the cumulated PDRM rate corresponding to the presence of mutations in blood and/or in CVF was 33.3%.

### 3.5. Longitudinal Follow-Up after ART Initiation

**Figure 2:**
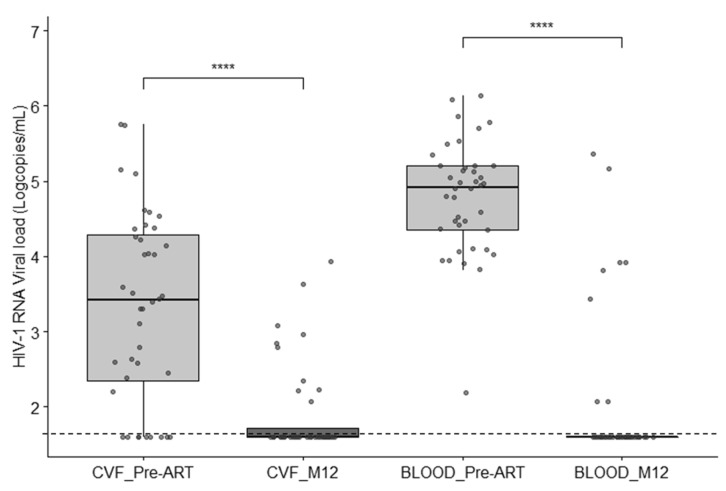
Scatter/box plot showing the viral loads in CVF and blood before and after 12 months of ART; **** *p* < 0.0001. Dashed line: threshold of viral load detection.

From the 52 females (66.7%) still present for follow-up at month 12, all of them gave blood and 40 gave CVF. A significant decrease in VL in blood and CVF (−2.88 ± −1.11 and −1.76 ± −1.40 log_10_ copies/mL, respectively; *p* < 0.0001) was observed between day 0 and M12 (Figure 2). An undetectable HIV RNA VL in blood was reached in 84.6% of patients after 12 months of ART. At month 12 after ART initiation, 10 out of 40 (25%) CVF samples remained positive for HIV-1 RNA detection (median, 2.84 log_10_ copies/mL, range (2.07–3.94)). In seven females (17.5%), HIV RNA was detected in CVF but not in the corresponding blood sample. The mean quantity shed in these last seven was 3.02 log_10_ copies/mL. No correlation was observed between HIV RNA viral loads in blood and CVF in post-treated patients (*r* = 0.26, *p* = 0.11). A genotyping profile was successfully obtained in four out of the 10 CVF samples positive for HIV VL. No difference in terms of resistance profiles was observed compared to the first CVF sample (no mutations in three cases and K103N in one). A persistent shedding of HIV RNA in CFV was not correlated to the clinical stage, the viral load in blood or CVF at the time of ART initiation, or the presence of PDRMs in blood or CVF. 

### 3.6. Vaginal Microbiota Analysis

**Figure 3:**
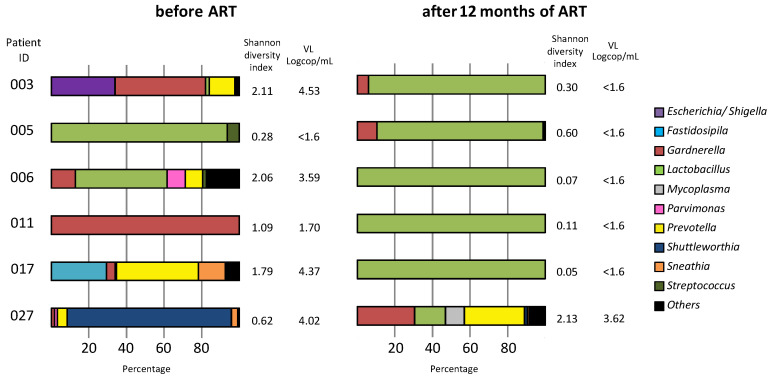
Cervicovaginal microbiota analysis in six patients according to HIV viral load in CVF, before and 12 months after ART initiation.

The study of the cervicovaginal microbiota was done in six females at entry and after 12 months of ART (Figure 3). A *Lactobacillus*-dominated microbiota was detected in CVF samples, exhibiting undetectable HIV-1 viral load before (female 005) and after 12 months of ART (females 003, 005, 006, 011, and 017). A higher diversity of bacteria including bacterial vaginosis (BV)-associated organisms (*Gardnerella*, *Prevotella*, and *E*. *coli*) and a paucity of *Lactobacillus* were detected in samples exhibiting detectable HIV-1 RNA before (003, 006, 011, 017, and 027) and after 12 months of ART (027).

## 4. Discussion

At the time of large-scale use of ART in RLCs, its efficacy on the HIV VL in the cervicovaginal compartment has been poorly investigated. Our work aimed to quantify the viral shedding in CVF before and after 12 months of first-line ART in females newly diagnosed positive for HIV-1 in Bamako. First, we bring some arguments in favor of a compartmentalization of HIV-1 in the genital tract of some females before ART initiation, evidenced by an absence of correlation between HIV-1 viral loads in blood and CVF, as well as discrepancies in terms of RT/protease gene sequence homology and of PDRM patterns between these two compartments. Regarding PDRM discrepancies between blood and CVF, it cannot be excluded that missed mutations were present on some minority strains not detected by the Sanger sequencing protocol used in our study. Further investigations are needed to check more precisely the compared HIV-1 variant mixture in both compartments, notably using an NGS approach. As a whole, a so-called “cumulated PDRM rate”, taking into account blood and CVF results, can be estimated as high as 33.3% in our population. Some of these mutations were polymorphic; the nonpolymorphic ones, namely, V106I, E138A, and V179E, could also compromise the use of second-generation NNRTIs such as etravirine or rilpivirine, according to current algorithms. These specific DRMs were also observed by others in untreated people or in patients failing first-line ART [24,25,26]. In our work, the presence of mutations at positions 106, 138, and 179 may be more likely related to natural variability and founder effects than to the use of etravirine or rilpivirine that were not available in Mali at the time of the study. These mutations could also have deleterious consequences in the case of first-line treatment based on second-generation NNRTIs since there is no routine resistance testing of treatment-naïve patients in Mali. The recent introduction of dolutegravir in Mali in 2020 could contribute to overcome the increase of primary resistance to NNRTI. Indeed, this integrase inhibitor could play a major role in sub-Saharan Africa because of its high barrier to resistance, good tolerability, and low cost, despite its described neural toxicity in infants when used in women at the time of conception.

After 12 months of ART, 17.5% of females remained positive for HIV RNA in the genital tract, whereas their viremia was undetectable. This is in agreement with the study of Low et al., conducted in 188 African sex workers under ART for at least 8 years, which reported a cervicovaginal shedding in 16% of them without detectable HIV-1 VL at the blood level [27]. This residual rate of positivity in the genital compartment is higher than that reported recently in a large cohort of African females after 12 months of ART (4.6%) [28], and it is in contradiction with former studies indicating no vaginal viral shedding in women exhibiting blood VL suppression [29,30]. These discrepancies may be explained, at least in part, by differences in terms of antiretroviral regimen and of therapy duration, which was only 12 months in our case. A previous report showed a decline in HIV RNA shedding in the female genital reservoir up to 18 months after ART initiation [31].

In our experience, the mean HIV VL in the genital tract was 3.12 log_10_ copies/mL, corresponding to a risk of two transmissions per 100 person-years (95% CI: 0.2 to 4.9), according to Beaten et al., who asserted that the relationship between genital VL and transmission risk was independent of plasma HIV-1 concentrations [16]. This persisting local replication of HIV may be due to combined factors including poor diffusion of ARV in the genital tract, oral contraceptive use, local inflammation, and the presence of bacterial vaginosis or other STIs.

After evaluation of the microbiota composition in a very limited number of CVF samples before and after 1 year of ART, we detected in six females a vaginal dysbiosis in HIV RNA-positive samples, with a further restoration of *Lactobacillus*-dominant microflora under ART. *Lactobacillus* spp. are the most common commensal bacteria of the human vagina, and their depletion of the vaginal microbiota is commonly associated with high-risk sexual behaviors and a high acquisition rate of STIs, including HIV [32,33,34]. The association between vaginal dysbiosis and detection of residual HIV RNA in the female genital tract despite blood VL suppression under ART is an additional argument for the systematic screening and treatment of STIs and genital infections with the aim of decreasing local inflammation and the risk of HIV-1 transmission, especially in the context of high-level PDRM areas. Of note, altering the vaginal microbiome is one of the new directions being pursued in HIV patient care, notably by using protonated-lactic-acid-based microbicides or *Lactobacillus*-based probiotics aimed at reducing the prevalence of bacterial vaginosis, thereby lowering the risk of acquiring or transmitting HIV [14,15,35].

As already pointed out by Beaten et al. [16], our findings also question the fact that blood VL may be the most important predicting factor of vaginal HIV-1 shedding, and that the U = U rule can be universally applied. In our opinion, caution should still be applied, especially in RLCs, when using blood viral load as a predictive factor of sexual transmission, despite very reassuring results obtained in large clinical trials such as HPTN 052 and Partner 2, demonstrating that effective ART, associated with the stable suppression of PVL, could actually suppress the risk of HIV transmission [7,36]. Indeed, if STIs were associated with HIV-1 genital shedding in the work of Low et al. [27], the opposite was found in Partner 2 study, outlining the importance of the studied population (gay couples in Europe [36] versus heterosexual women from Burkina Faso [16]), the sampling, and the possibility of an intermittent viral shedding at the genital level. Therefore, the U = U concept, which is commonly validated for men having sex with men in high-resource countries, may need to be nuanced in women from RLC who do not benefit from regular medical surveillance, notably in terms of genital infections.

Our work was done under real-life conditions, which explains some limitations including the size of the studied population, the high rate of LTFU, and the limited number of samples successfully analyzed for ARV resistance mutations and microbiota composition. We also were not able to check the persistence of HIV-1 genital strains over time and their infectivity. However, our findings emphasize the urgent needs of (i) reinforcing education to improve the retention of women in care system, (ii) regular virological monitoring before and during ART, (iii) improving surveillance of genital infections and dysbiosis with appropriate therapy, and (iv) reinforcing the survey of PDRMs in RLCs. These objectives are important prerequisites to the success of the UNAIDS 95/95/95 goals for eradicating the AIDS epidemic in Africa.

## Figures and Tables

**Table 1:** Paired resistance genotyping profiles successfully obtained in blood and CVF samples from 24 females before initiation of ART.

ID	Age (Years)	Clinical Stage (CDC)	CD4^+^ Cells/ mm^3^	Genotype	VL at Entry (Copies/mL)		Resistance-Associated Mutations *Polymorphic*		Drug Resistance (Stanford HIV db Program Rules)		Homology of Sequence between Blood and CVF Strains (%)
					Blood	CVF	Blood	CVF	Blood	CVF	
001	43	I	988	CRF02_AG	87,200	1250	None *K20I M36I H69K L89M*	None *K20I M36I H69K L89M*	None *None*	None *None*	92.2
002	37	III	9	CRF02_AG	49,200	380	None *K20I M36I H69K L89M*	L74V *K20I M36I H69K L89M*	None	ABC (intermediate)	93.6
003	52	III	103	CRF26_AU	163,000	34,000	None *L10I/V M36I H69Q L89M*	None *L10I M36I H69Q L89M*	None	None	99.2
010	25	I	844	CRF02_AG	162,000	166	None *K20I M36I H69K L89M*	None *K20I M36I H69K*	None	None	96.9
011	29	I	1405	B	50,000	50	None *None*	None *None*	None	None	97.6
014	35	III	158	CRF02_AG	225,000	120,000	None *K20I M36I H69K L89M*	None *K20I M36I H69K L89M*	None	None	100
019	46	III	53	CRF02_AG	1,620,000	14,200	E138A *L10V K20M/V M36I H69K L89M*	E138A *L10V K20M/V M36I H69K L89M*	RPV (low level) ETR (potential low level)	RPV (low level) ETR (potential low level)	99.7
025	34	III	293	CRF02_AG	325,000	142,000	None *K20I M36I H69K L89M*	None *K20I M36I H69K L89M*	None	None	100
026	35	II	427	CRF02_AG	1,200,000	3900	None *K20I M36I H69K L89M*	None *K20I M36I H69K L89M*	None	None	98.8
030	28	III	68	CRF02_AG	468,000	67,500	None *L10V K20I M36I H69K L89M*	None *L10V M36I H69K L89M*	None	None	98.8
041	49	II	357	CRF02_AG	10,700	13,700	None *K20I M36I H69K L89M*	None *K20I M36I H69K L89M*	None	None	97.5
044	40	III	209	CRF02_AG	140,000	250	None *K20I M36I H69K L89M* *V179I*	D67N *K20I M36I H69K L89M* *V179I*	None	ZDV (low level)	98.2
046	33	III	113	CRF02_AG	300,200	5000	K103N *M36I H69K L89M*	None *M36I H69K L89M*	None	None	90.9
047	40	III	165	CRF02_AG	28,500	67,500	None *L10V M36I H69K L89M*	D67N *L10V M36I H69K L89M*	None	ZDV (low level)	98.1
049	29	I	436	CRF02_AG	33,000	282	None *K20I M36I H69K L89M*	None *K20I M36I H69K L89M*	None	None	91.3
055	46	II	412	CRF02_AG	142,000	37,700	None *L10V K20I M36I H69K L89M*	None *L10V K20I M36I H69K L89M*	None	None	99.1
070	26	IV	180	CRF02_AG	8804	16,500	None *K20I M36I H69K L89M*	None *K20I M36I H69K L89M*	None	None	99.3
080	47	I	536	A1	100,000	26,400	None *K20I M36I H69K L89M*	None *K20I M36I H69K L89M*	None	None	98.2
081	34	IV	110	CRF02_AG	4300	27,400	None *L10V K20I M36I H69K L89M* *V179I*	None *K20I M36I H69K L89M*	None	None	93.3
085	32	II	592	CRF02_AG	29,500	575,000	V179E *K20I M36I H69K L89M*	None *M36I H69K L89M*	EFV (potential low level) NVP (potential low level) ETR (potential low level) RPV (potential low level)	None	93.4
091	60	II	183	CRF06_cpx	7950	41,400	V106I *K20I M36I H69K L89M*	V106I *K20I M36I H69K L89M*	DOR (low level) NVP (potential low level) ETR (potential low level) RPV (potential low level)	DOR (low level) NVP (potential low level) ETR (potential low level) RPV (potential low level)	96.3
096	33	II	225	CRF06_cpx	758,000	23,440	None *L10V K20I M36I H69K L89M*	V179E *L10V K20I M36I H69K L89M*	None	EFV (potential low level) NVP (potential low level) ETR (potential low level) RPV (potential low level)	95.2
101	25	III	13	CRF02_AG	241,000	13,200	None *L10V K20I M36I H69K L89M*	None *L10V K20I M36I H69K L89M*	None	None	100
107	18	II	787	CRF02_AG	19,500	8300	None *K20I M36I H69K L89M*	None *K20I M36I H69K L89M*	None	None	100

## Data Availability

Nucleotide sequences were deposited in GenBank.
